# Lico A Causes ER Stress and Apoptosis via Up-Regulating miR-144-3p in Human Lung Cancer Cell Line H292

**DOI:** 10.3389/fphar.2018.00837

**Published:** 2018-07-31

**Authors:** Gang Chen, Yueping Ma, Zhe Jiang, Yuan Feng, Yueqing Han, Yetian Tang, Juan Zhang, Hui Ni, Xuezheng Li, Ning Li

**Affiliations:** ^1^School of Traditional Chinese Materia Medica, Shenyang Pharmaceutical University, Shenyang, China; ^2^State Key Laboratory for Chemistry and Molecular Engineering of Medicinal Resources, Guangxi Normal University, Guilin, China; ^3^Department of Pharmacy, Affiliated Hospital of Yanbian University, Yanji, China; ^4^XinJiang Institute of Chinese Materia Medica and Ethnodrug, Ürümqi, China

**Keywords:** autophagy, apoptosis, CHOP, miR-144-3p, homology modeling, docking

## Abstract

During our study on the bioactivities of natural flavonoids, we found that the total flavonoids (TFs) and the main constituent of it, licochalcone A (lico A), activated unfolded protein response (UPR) and induced autophagy and thereby apoptosis in H292 cells. MicroRNAs, such as the tumor repressor miR-144-3p, were reported to be differentially expressed in lung cancer cells and were linked to ER stress, autophagy, and apoptosis. However, the underlying miRNA-based mechanism for lico A modulating proliferation, autophagy and apoptosis in lung cancer cells is elusive. In this study, we found that miR-144-3p was down-regulated in H292 cells comparing to normal embryonic lung cells WI-38, and lico A (10 μM) could increase miR-144-3p level in H292 cells. Knockdown of miR-144-3p significantly abrogated the apoptosis and proliferation-inhibiting effects of lico A, and lico A could enhance the proliferation-inhibiting effect and apoptosis induced by miR-144-3p overexpression. Moreover, overexpression miR-144-3p could induce ER stress by down-regulating Nrf2, and lico A enhanced the Nrf2 down-regulation caused by miR-144-3p overexpression. Co-transfection experiments showed that lico A potentially increased the dicing of pre-miR-144 so as to increase the mature miR-144-3p level. Interestingly, high level of lico A (40 μM) up-regulated CHOP protein, but failed to increase the downstream genes levels of CHOP, including Bim and Bcl-2 in H292 cells. Docking studies indicated that CHOP-mediated pathway was potentially blocked by high dose of lico A. Our results suggested that lico A could cause UPR, autophagy and apoptosis, and the underlying mechanism involved up-regulation of miR-144-3p, and increased lico A level would also increase the potential for lico A inhibiting CHOP-dependent apoptosis in H292 cells.

## Introduction

MicroRNAs (miRNAs) have been reported to be differentially expressed in human cancer cells and are linked to oncogenesis of various cancers. For example, miR-144-3p is critical for air pollution-related lung tumorigenesis and is the most significantly down-regulated miRNA in patients of non-small cell lung cancers (NSCLCs) (Pan et al., 2015). Researches have proved that miR-144-3p can target many oncogenic factors, including VEGF, c-MET, TIGAR, etc. (Chen et al., 2015; Lan et al., 2015; Tao et al., 2015), to suppress proliferation and induce apoptosis in tumor cells.

As opposed to synthetic molecules that are designed to interact with only one or a few of certain protein targets, natural products often exhibit multi-bioactivities by targeting various pathways and factors in the same time. How could that be achieved? This multi-target feature of natural products is typically the same as that of the non-coding RNAs. Non-coding RNAs, such as long non-coding RNAs (lncRNAs) and miRNAs (Xu et al., 2017; Yan et al., 2017), can also inversely target many factors in different type of human organs simultaneously. Depending on the basal level, which varies in different organs, one miRNA could show different bio-function. Some studies showed that natural products can be able to modulate non-coding RNAs so as to target many pathways simultaneously (Liu et al., 2018). Though there are a few of research papers reporting the relationship between natural molecules and miRNAs, for most majority of miRNAs, their relationships to natural products are still elusive. For example, so far, little is known about the effect and mechanism of natural products modulating miR-144-3p which was found to be down-regulated in lung cancer cells and tissue in this paper.

Licochalcone A (lico A) is capable of induce ER stress, autophagy, and apoptosis in cancer cells (Yuan et al., 2013; Xue et al., 2018). However, the anti-tumor effect of lico A on H292 cells and the underlying miRNA-based mechanism remain unknown. In our studies explaining the miRNA-based mechanism for natural products, we verified several lung cancer-related miRNAs could be modulated by lico A, such as mir-20a (data not shown) and miR-144-3p, etc. In present study, the antitumor effect of lico A and the relationship between lico A and miR-144-3p in H292 cells were discussed.

## Materials and Methods

### Preparation of the Total Flavonoids and Lico A From Licorice

The total flavonoids (TF) and lico A used in the experiment were prepared according to the method described in our previous work (Li et al., 2015).

### Cell Culture

H292 and WI-38 cells were purchased from American Type Culture Collection (Manassas, VA, United States) study. Cells were cultured (37°C and 5% CO_2_) in appropriate medium (DMEM with 10% FBS) supplemented with 100 μg/ml streptomycin, 100 U/ml penicillin and 2 mM glutamine.

### Real-Time Fluorescence Quantitative Polymerase Chain Reaction Analysis

Quantitative real-time PCR was performed to quantitate atg genes and miR-144-3p, using SYBR Premix EXtagII (TaKaRa, Dalian, China) in the PRISM 7900HT system (Applied Biosystems, Carlsbad, CA, United States). For miR-144-3p quantification: Bulge-loop miRNA qRT-PCR Primer Sets (one RT primer and a pair of qPCR primers for each set) specific for miR-144-3p is designed by RiboBio (Guangzhou, China). U6 small nuclear RNA (snRNA) or GAPDH for atg genes was used as endogenous control. Total RNA was isolated from cells using an RNeasy Mini Kit (Qiagen, Valencia, CA, United States). RT-PCR for Atg1, Atg3, Atg5, Atg6, Atg8, Atg14, Atg16, Atg17 were performed using ABI TaqMan Gene Expression Assays (Applied Biosystems, Foster City, CA, United States). RNA was reverse transcribed by using the high-capacity cDNA archive kit (Applied Biosystems, United States). RT-PCR and subsequent calculations were performed by the Step One Plus Real-time PCR system (Applied Biosystems, United States), which detected the signal emitted from fluorogenic probes during PCR. All samples were analyzed three times. The RT-qPCR results were expressed relative to miR-144 expression levels at the threshold cycle (Ct), which were then converted to fold change (2^-ΔΔC_t_^).

### Transfection With miRNAs

Pre-miR-144 and miR-144 inhibitor were purchased from Ambion (Austin, TX, United States). Untreated H292 cells, growing exponentially, were plated at 2 × 10^7^/well in 2.5 ml DMEM medium for 24 h on six-well plates. Once cells reached about 50% confluence, transfection was conducted. Lipo2000 from Invitrogen (Carlsbad, CA, United States) was used in all transfection processes according to the manufacturer’s instructions. Each cell line was separated into three groups: the non-transfected blank group (blank); scrambled miR-144 transfected negative control group (scramble); and the pre-miR-144 or miR-144 inhibitor transfected group.

### Western Blot Analysis

Western blot analysis was performed as previously described (Chen et al., 2017). H292 cells were pre-treated with total flavones or lico A for 24 and 48 h. Cells were washed with ice-cold PBS and lysed with lysis buffer. The protein content was determined by BCA assay. Equal amounts of protein and the Precision Plus Protein Standards (Bio-Rad) were resolved by SDS-PAGE gels (12% gel for caspase 3 and 8, Bim, Bax, LC3 and 10% gel for all other proteins) and transferred onto Immobilon-P membranes (Millipore). After soaking in blocking buffer, the membrane was incubated overnight with primary antibodies, followed by horseradish peroxidase-conjugated secondary antibodies. BiP, CHOP, phosphorylated (p-)eIF2a, p-PERK, p-activating transcription factor (ATF) 4, Bim, Bax, Bcl2 (Cell Signaling Technology, Beverly, MA, United States); Nrf2, and r-GSSC (Santa Cruz Biotechnology); anticaspase 3 (Chemicon International, Billerica, MA, United States); and rat anticaspase 8 (mouse specific) (AlexisBiochemicals, San Diego, CA, United States), which detects both procaspase 8 and cleaved caspase 8; Atg1 (biorbyt); Atg3 (Abcam); Atg16 (Abbiotec); Atg6 (Novus); Atg16 (Abbiotec); Membranes were exposed to goat anti-rabbit or anti-mouse (Jackson ImmunoResearch Laboratories, West Grove, PA, United States) secondary anti-bodies. An antibody against β-actin or GAPDH (Santa Cruz Biotechnology) served as an endogenous reference.

### Luciferase Reporter Assay

The human Nrf2 3′ untranslated region (UTR) fragment containing putative binding sites for miR-144-3p was amplified by PCR from human genomic DNA. The mutant 3′-UTRs were obtained by overlap extension PCR. The fragments were cloned into a pmirGLO reporter vector (Promega, Madison, WI, United States), downstream of the luciferase gene, to generate the recombinant vectors pmirGLO-WT and pmirGLO-MUT. For the luciferase reporter assay, H292 cells were co-transfected with miRNA (pre-miR or scrambled-miR negative control) and reporter vectors (pmirGLO-WT reporter vectors or pmirGLO- MUT reporter vectors), using lipo2000. Luciferase activities were measured with a Dual-Luciferase assay kit (Promega, Madison, WI, United States) according to manufacturer’s instructions at 24 h post-transfection. Experiments were repeated three times in triplicate.

### Cell Growth Assay

Cell Counting Kit (CCK8; Dojindo, Japan) was used to measure cell proliferation. Cells were cultured in 96-well plates (100 μl/well) in complete DMEM and were maintained in an incubator at 37°C and 5% CO_2_ CCK solution (10 μl) was added to each well. Optical density (OD) value was measured at 490 nm (OD_490_) to estimate viable cell number. The assay was repeated in three-independent experiments.

### Flow Cytometry Assay

Annexin V/PI staining assay was employed to determine apoptosis, and apoptosis analysis was carried out using an apoptosis detection kit (Keygen, Nanjing, China) according to the manufacturer’s instructions. Briefly, 1 × 10^6^ cells/well of H292 were cultured in a 6-well plate. The next day, the cells were treated with total flavones or lico A and incubated for 24 h. At indicated times, cells were harvested, and both attached and floating cells were collected, then washed twice with ice-cold PBS, and resuspended in 100 μL binding buffer that contained Annexin V and PI for 15 min at 37°C in the dark. The numbers of healthy viable cells, apoptotic and necrotic cells were measured by flow cytometry (Becton Dickinson, Franklin Lakes, NJ, United States) and analyzed using Cell Quest software. The apoptosis rate was given by the following formula:

Apoptosis rate % = (number of apoptotic cells)/(number of total cells observed) × 100%.

### Docking

The sequence of basic region leucinzipper (BRLZ) of CHOP was subjected to blast so as to search the template for homology-modeling with Schrodinger 2013 (Chen et al., 2017) software package. The results showed that the sequence of 1NWQ gave the best result in this blast-based template searching (Score: 33.5, similarity: 31.1475, length: 54). The structure for BRLZ was finally established with homology-modeling method provide in the Schrodinger 2013 software package with default setting. Before docking, the preparations of BRLZ and lico A conducted following the standard protocol of the Protein Preparation and Ligprep Wizards, respectively, of the (Chen et al., 2017) Suite. Docking studies were implemented via Grid-based Ligand Docking with Energetics (Glide) method in XP (Extra-Precision) mode that can give more precise G Score, by which the docking results were ultimately ranked.

## Results

### Total Flavone (TF) and Lico A Inhibited Cell Proliferation, and Induced ER Stress as Well as Apoptosis in H292 Cells

**FIGURE 1:**
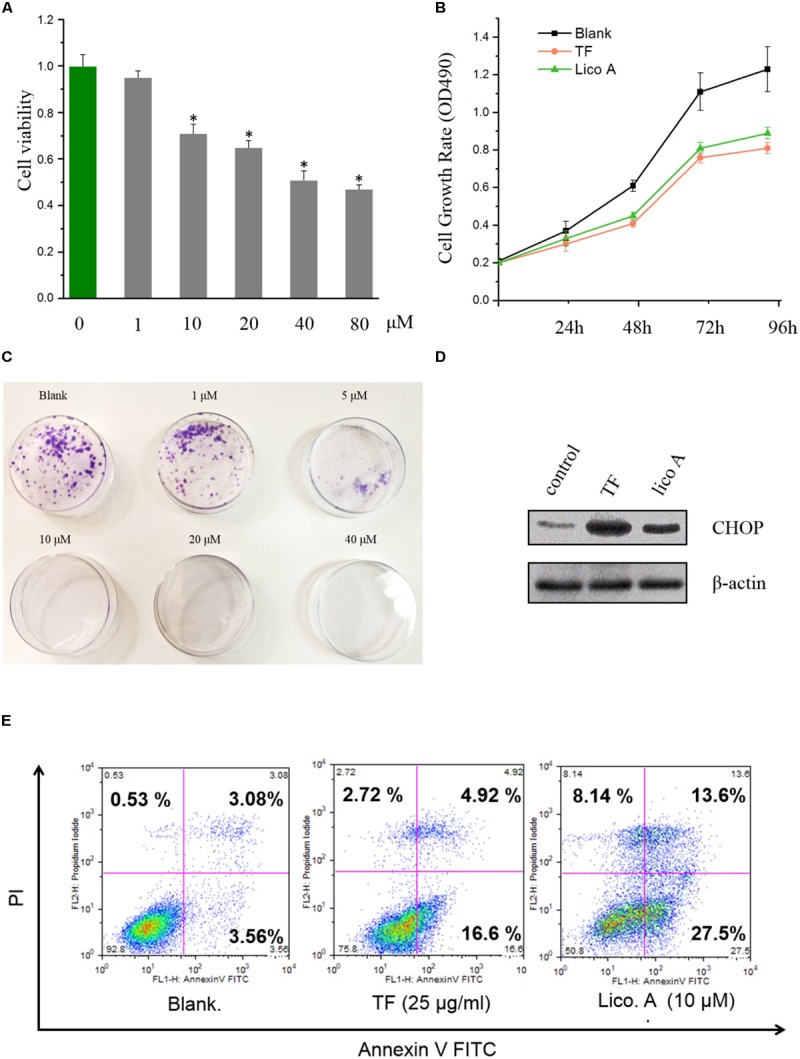
Total flavone (TF) and lico A inhibite cell proliferation and induced ER stress as well as apoptosis in H292 cells. **(A)** Cell viabilities of H292 cells treated with lico A of different concentrations (^∗^*P* < 0.05 referenced to 0 μM group). **(B)** The cell growth rates (OD_490_) of total flavone (TF) and lico A groups go slower than the blank group. **(C)** Lico A represses H292 cell colon formation. **(D)** ER-stress response protein CHOP is up-regulated by TF (25 μg/ml) and lico A (10 μM). **(E)** Apoptosis rates of TF (25 μg/ml) and lico A (10 μM) groups are higher than blank group.

The inhibitory effect of lico A on H292 cells were evaluated with CCK8 method, and the results showed that lico A could suppress H292 cell viability dosage-dependently within the concentration ranging from 10 to 40 μM (**Figure 1A**). Also, comparing to blank group, the OD_490_ values for total flavone (TF) group from *Glycyrrhiza uralensis* F. and lico A (10 μM) group were all significantly decreased (*P* < 0.05) in H292 cells (**Figure 1B**). Moreover, lico A (5 μM) potently suppressed the colon formation of H292 cells, and 10–40 μM lico A totally inhibited H292 cells from colon formation (**Figure 1C**). Thus, we chose 10 μM lico A in the following experiment unless otherwise indicated. We then examined the level of ER-stress response protein CHOP via western blot analysis, and the outcomes of which proved a significant enhancement of CHOP expression induced by TF and lico A at 48 h, suggesting the occurrence of ER stress in H292 cells (**Figure 1D**). Furthermore, the flow cytometry experiment indicated that apoptosis rates in TF and lico A groups were higher than those in the blank group (**Figure 1E**). As lico A was the main constituent in the total flavone (around 7% of the total flavone), the effect of TF was thus attributed to lico A.

### Lico A Increased miR-144-3p Level in H292 Cells

**FIGURE 2:**
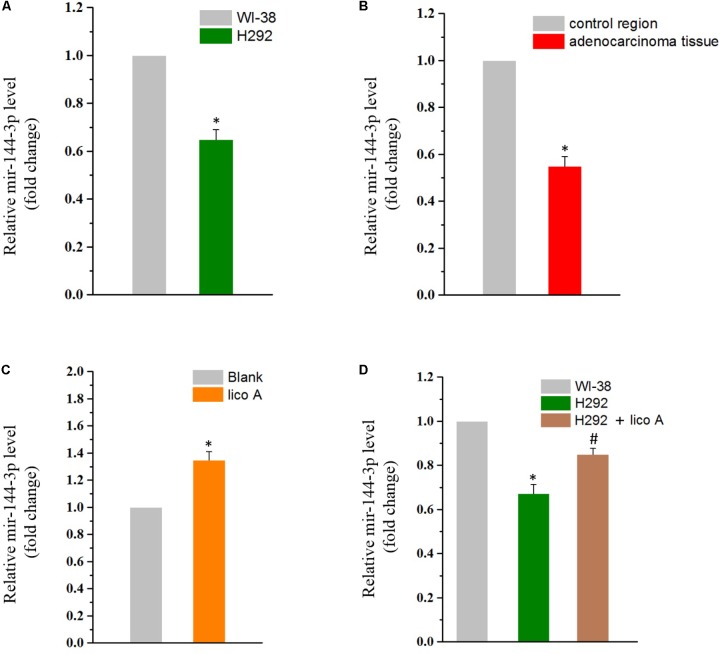
**(A)** MiR-144-3p is down-regulated in H292 cells comparing to WI-38 cells (^∗^*P* < 0.05 referenced to the WI-38 group). **(B)** MiR-144-3p is down-regulated in adenocarcinoma tissues comparing to in the control region of NSCLC patients (^∗^*P* < 0.05 referenced to the control region). **(C)** Lico A (10 μM) increases miR-144-3p level in H292 cells (^∗^*P* < 0.05 reference to the blank group). **(D)** The miR-144-3p level is close to that of the WI-38 cells in 10 μM lico A-treated H292 cells (^∗^*P* < 0.05 referenced to the WI-38 group; ^#^*P* < 0.05 referenced to the H292 group).

Calculating relative miR-144 concentrations by fold changes, we found that the miR-144-3p expression was lowered in H292 cells than in normal embryonic lung WI-38 cells (**Figure 2A**). This decline in the miR-144-3p level was also observed in patients with non-small cell lung cancer (NSCLC), who’s adenocarcinoma tissue exhibited much higher level of miR-144-3p than the those from the control region (**Figure 2B**). We then tested if lico A would affect miR-144-3p level in H292 cells. The result showed that lico A could increase miR-144-3p level (**Figure 2C**), making it more close to that in WI-38 cells (**Figure 2D**).

### Knockdown of miR-144-3p Reversed the Proliferation Inhibition, ER Stress, and Apoptosis Induced by Lico A

**FIGURE 3:**
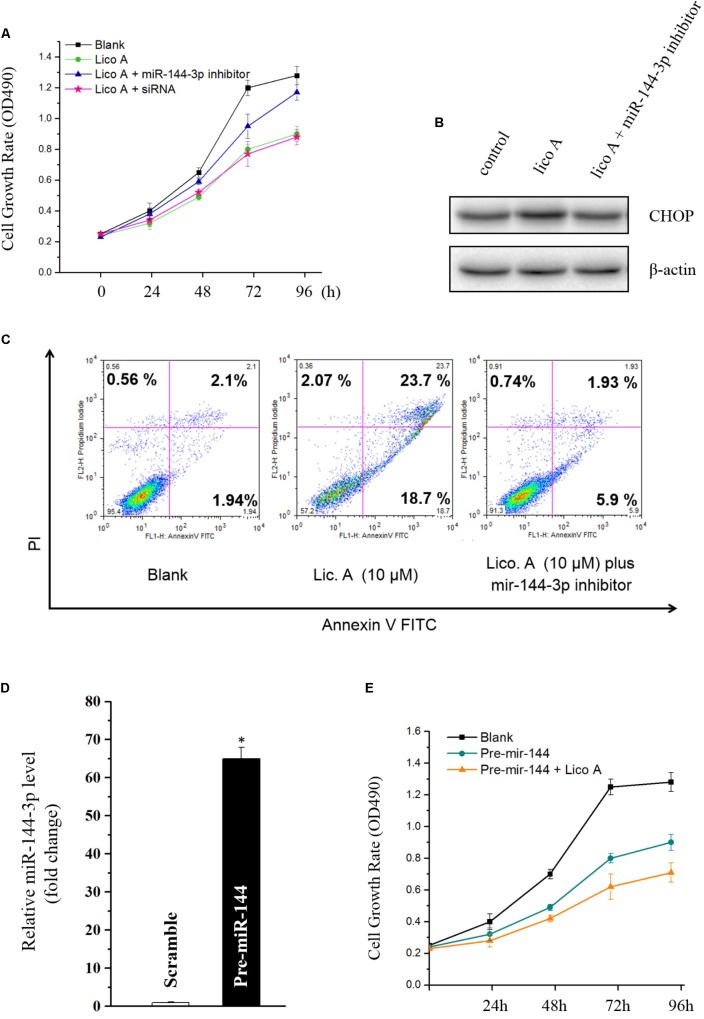
**(A)** Knockdown of miR-144-3p by miR-144-3p inhibitor/siRNA enhances lico A suppressing H292 cell proliferation. **(B)** The up-regulation of CHOP by lico A is reversed by miR-144-3p inhibitor. **(C)** Lico A-induced apoptosis is also attenuated by miR-144-3p inhibitor. **(D)** MiR-144-3p is overexpressed by pre-miR-144 transfection (^∗^*P* < 0.05 referenced to the scramble group). **(E)** Overexpression of miR-144-3p suppresses H292 cell proliferation and lico A synergistically strengthens the suppressive effect of the overexpressed miR-144-3p.

To identify whether miR-144-3p would affect lico A inducing ER stress, apoptosis, and inhibition on cell proliferation in H292 cells, miR-144-3p was knockdown via transfection of either the miR-144-3p inhibitor or the siRNA. The results showed that reduced miR-144-3p level substantially attenuated the inhibition of lico A on both cell proliferation and colon formation (**Figure 3A**). Western blot results revealed that decrease of miR-144-3p totally reversed the lico A-induced CHOP dysregulation (**Figure 3B**). Moreover, the flow cytometry experiment showed that apoptosis caused by lico A was also severely interfered with by the decrease of miR-144-3p (**Figure 3C**). Subsequently, we examined if lico A would enhance miR-144-3p inhibiting H292 cell proliferation. The miR-144-3p was overexpressed by transfecting pre-miR-144, which is the precursor of miR-144-3p. And the results showed increased miR-144-3p potently suppress H292 cell proliferation, and co-transfection of lico A with pre-miR-144 strengthened the inhibitory effect of miR-144-3p in H292 cells (**Figures 3D,E**).

### Lico A Attenuated Nrf2 Levels via Increasing miR-144-3p Level in H292 Cells

**FIGURE 4:**
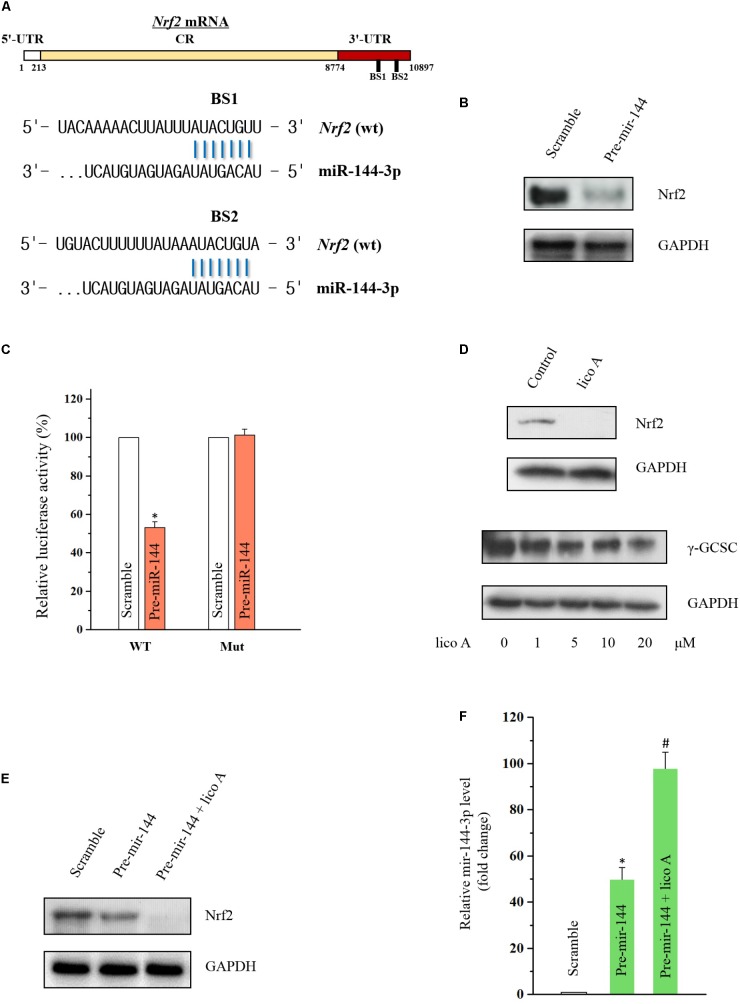
Lico A decrease Nrf2 expression via miR-144-3p modulation in H292 cells. **(A)** Predicted binding sites of Nrf2 mRNA for miR-144-3p. **(B)** Overexpressed miR-144-3p by transfection of pre-miR-144 decreases Nrf2 level. **(C)** Luciferase results for miR-144-3p interacting with 3′-UTR of Nrf2 mRNA (WT: entire 3′-UTR of Nrf2 mRNA; MUT: sequence of the binding sites deleted based on the sequence of WT; ^∗^*P* < 0.05). **(D)** Lico A (10 μM) attenuates Nrf2 expression and the Nrf2 down-stream gene γ-GCSC. **(E)** Lico A (10 μM) synergistically enhances the Nrf2 suppression caused by miR-144-3p overexpression. **(F)** MiR-144-3p level is higher in the co-transfection group of lico A (10 μM) and pre-miR-144 than that in the pre-miR-144 transfection group (^∗^*P* < 0.05 referenced to the scramble group; ^#^*P* < 0.05 referenced to the pre-miR-144 group).

In order to further examine the effect of lico A modulating downstream genes of miR-144-3p, the Nrf2 level was tested by western blot. Nrf2 was reported to be the direct target of miR-144-3p in hepatocellular carcinoma cell lines (Zhou et al., 2016), but the relationship between miR-144-3p and Nrf2 in lung cancer is still elusive. There are two predicted binding sites in the 3′-UTR of Nrf2 mRNA for miR-144-3p (**Figure 4A**). Thus, we overexpressed miR-144-3p via transfection of pre-miR-144 in H292 cells, and we observed that overexpressed miR-144-3p dramatically decreased Nrf2 protein level (**Figure 4B**). Furthermore, co-transfection with pre-miR-144 significantly suppressed luciferase activity of the reporter containing the wild-type 3′-UTR of the Nrf2 mRNA (**Figure 4C**), indicating that miR-144-3p down-regulated Nrf2 expression via directly binding to the putative binding site of Nrf2 mRNA. In this line of thinking, we tested the effect of lico A on Nuclear factor E2-related factor (Nrf2) expression, and the western blot results revealed lico A also decreased Nrf2 and its down-stream γ-glutamylcysteine synthetase catalytic subunit (γ-GCSc) protein levels (Li et al., 2017) in H292 cell line (**Figure 4D**). To prove if lico A decreased Nrf2 expression via increasing miR-144-3p, first, co-transfection experiment of pre-miR-144 and lico A was conducted, and the results substantiated that lico A could enhance the decrease of Nrf2 protein level caused by miR-144-3p (**Figure 4E**). Second, RT-qPCR results showed that lico A could enhance the miR-144-3p overexpression by the pre-miR-144 transfection (**Figure 4F**). These results suggested lico A could lower the Nrf2 protein level via increasing miR-144-3p expression.

### High Dose of TF and Lico A Caused UPR and Autophagy Without Affecting Apoptosis-Related Factors in H292 Cells

**FIGURE 5:**
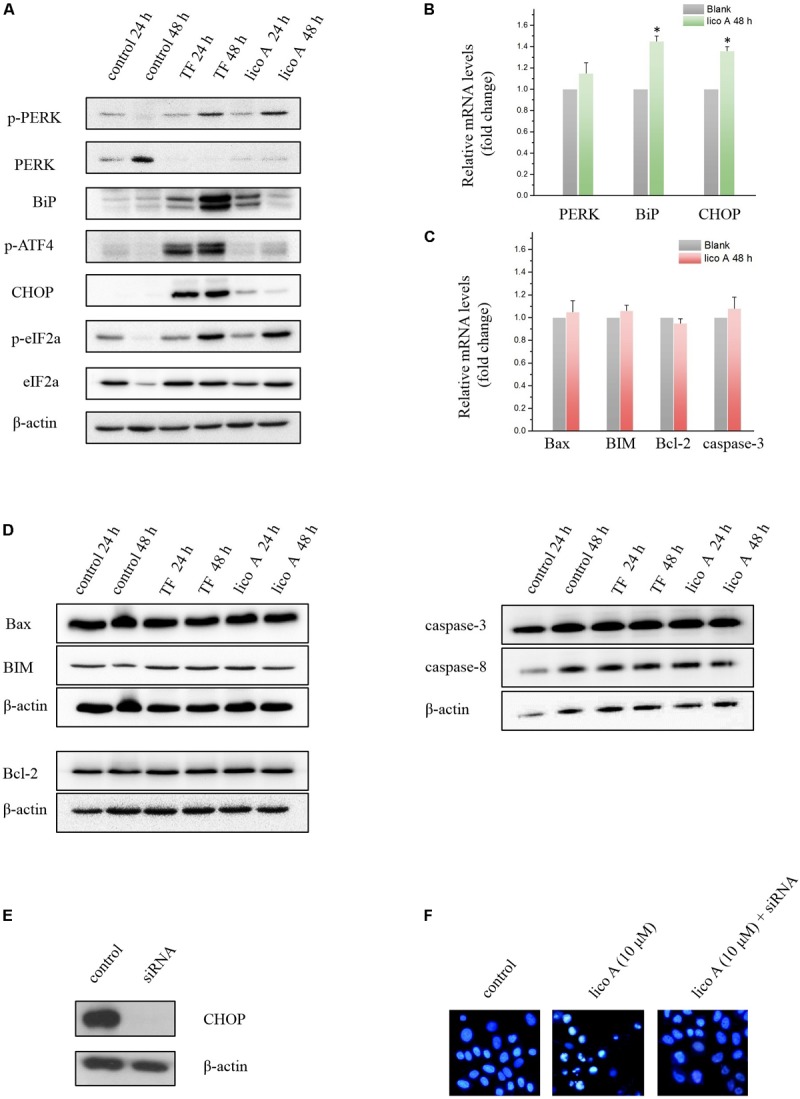
**(A)** Western blot results of ER stress-related factors affected by high dose of TF (75 μg/ml) and lico A (40 μM). **(B)** RT-qPCR results of ER stress-related factors affected by lico A (40 μM) (^∗^*P* < 0.05 referenced to the control group). **(C)** RT-qPCR results of apoptosis-related factors affected by lico A (40 μM). **(D)** High dose of TF (75 μg/ml) and lico A (40 μM) induce no significant aberrant expression of apoptosis-related factors in H292 cells. **(E)** CHOP expression is repressed by siRNA. **(F)** Apoptotic nuclear morphology was assessed by fluorescent DNA-binding dye Hoechst 33258. The 10 μM lico A-induced apoptosis (middle) is reversed by the siRNA for CHOP (right).

Western blot results showed that both TF (75 μg/ml) and lico A (40 μM) of high level enhanced the expression of bip and p-PERK levels at either 24 or 48 h, suggesting the initiation of UPR in H292 cells (**Figure 5A**). We then further examined the downstream ATF4, eIF2a, and CHOP levels. The results showed that the phosphorylation of ATF4 and eIF2a as well as the CHOP expression were all up-regulated at 48 h (**Figures 5A,B**), indicating that the PERK-ATF4-CHOP pathway was involved in the TF/licoA-induced UPR. However, the down-stream apoptotic genes of CHOP was not altered (**Figures 5C,D**). Then we silenced CHOP protein by siRNA (**Figure 5E**), and the results showed that 10 μM lico A induced apoptosis was reversed after the CHOP silencing in the nuclear staining experiment with Hoechst 33258 (**Figure 5F**).

**FIGURE 6:**
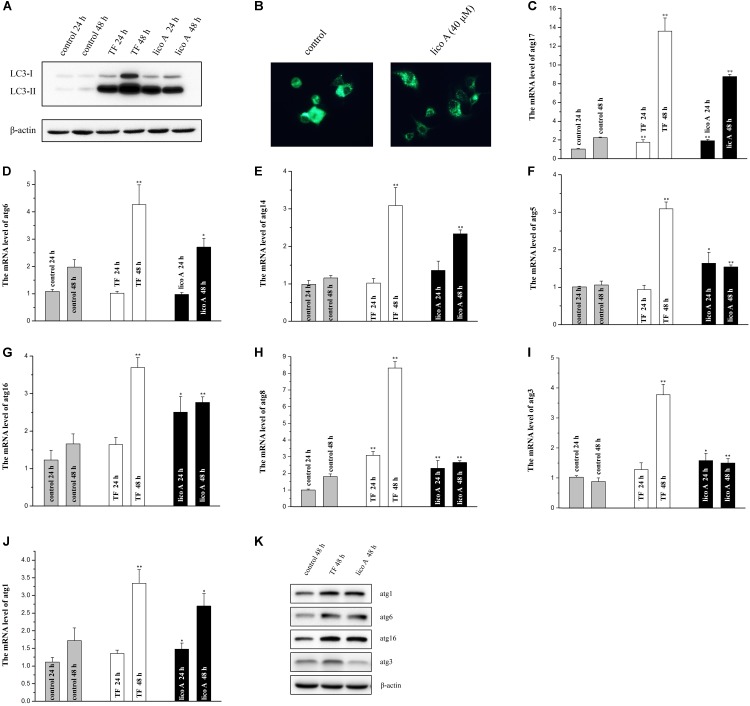
High dose of TF (75 μg/ml) and lico A (40 μM) trigger autophagy in H292 cells. **(A)** Western blot result for LC3-II accumulation. **(B)** The autophagy is detected with MDC staining. **(C–J)** RT-qPCR results for atg genes (^∗∗^*P* < 0.01 and ^∗^*P* < 0.05 referenced to the control group). **(K)** Western blot results for atg genes.

Driving CHOP transcription and therefore apoptosis, the enhancement of ATF4 phosphorylation also induces autophagy by transcriptionally regulating ATG genes (B’chir, 2013). The exact role of autophagy under ER stress is quite complicated and even controversial under different experimental circumstances. Most of the studies proved that autophagy can help coping with unfolded/misfolded proteins and therefore reducing ER stress, from which point of view being reckoned as cytoprotective or even organ protective (Ogata et al., 2006). However, prolonged and drastic autophagy may aggregate cell injury or even lead to cell death as well (Ciechomska et al., 2013). Therefore, the aforementioned up-regulation of ATF4 by lico A prompted us to investigate if the autophagy was also induced by TF and lico A. We first examined the LC3-II accumulation via western blot analysis, and the outcomes of which proved a significant enhancement of LC3-II accumulation induced by 40 μM lico A at both 24 and 48 h (**Figure 6A**). Subsequently the autofluorescent dye monodansylcadaverine (MDC) was to examine autophagy, and the results showed that 40 μM lico A could definitely induce autophagy in H292 cells (**Figure 6B**). Furthermore, we tested if TF and 40 μM lico A affected the transcription of ATG genes as ATF4 did after its phosphorylation. The results showed that mRNA levels of ATG1, ATG3, ATG5, ATG6, ATG8, ATG14, ATG16, and ATG17, along with the protein level of ATG1, ATG3, ATG6, and ATG16, were all increased by TF and lico A at 48 h (**Figures 6C–K**).

### Lico A Interacts With the Basic Region Leucinzipper of CHOP Protein and Thereby Blocks the CHOP Mediated Bcl-2/Bim Aberrant Regulation

**FIGURE 7:**
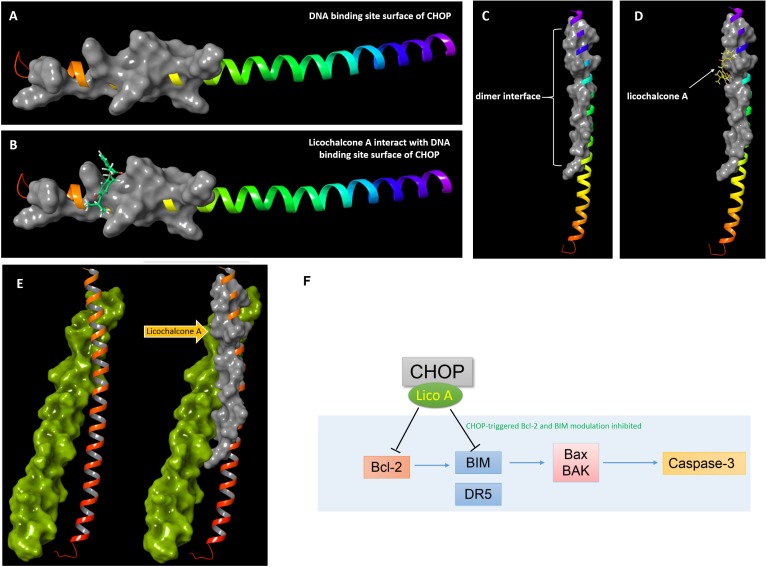
Docking results of lico A interacting with basic region leucinzipper of CHOP protein. **(A)** The DNA binding site surface of CHOP shown in gray cartoon representation with a solid surface. **(B)** Lico A represented in green color is docked into the DNA binding site surface of CHOP. **(C)** The dimer interface of CHOP shown in gray cartoon representation with a solid surface. **(D)** Lico A represented in yellow color is docked into the dimer interface of CHOP. **(E)** Protein-protein docking results of CHOP and C/EBPα. The C/EBPα was represented in yellow-green and the dimer-interface of CHOP was also represented in gray cartoon. **(F)** A proposed scheme for lico A inhibiting CHOP signaling pathway.

For we found silencing CHOP would reverse the apoptosis induced by 10 μM lico A, we wonder if high level lico A could inhibit CHOP due to the increased concentration so as to exert the same effect as the siRNA for CHOP. Thus, we focused on the basic region leucinzipper (BRLZ) of CHOP protein, where the dimer-interface and DNA-binding site locate, since blocking of CHOP protein would could prevent its down-stream apoptotic genes from being activated. As there is no CHOP crystal structure reported, we established the BRLZ moiety of CHOP via homology-modeling with (Chen et al., 2017) software package. The BLSAT search result showed that chain A of protein 1NWQ exhibited the best similarity (31.15%), comparing to BRLZ of CHOP that consists of 62 residues. So we used chain A of protein 1NWQ as the template to establish the BRLZ of CHOP by means of energy-based method provided in the package suite. The simulated structure was then subjected to protein refinement via following the protein refinement protocol in the package suite. Then we docked lico A into the refined BRLZ of CHOP to examine its potential ability to block the CHOP mediated signaling through occupying either the DNA interface, or the dimer interface of CHOP. Interestingly, lico A exhibited potent binding potentials in interacting with both the DNA-binding and dimer interfaces (**Figure 7**), which would consequently suppress the downstream BIM transcription and thereby down-stream apoptosis-related factors in H292 cells.

## Discussion

Lico A has been reported to be capable of suppressing tumor proliferation and interfering with the process of autophagy and ER stress. However, the question of whether the lico A-induced intervention on these cell functions involves miRNA-related mechanism has been kept unknown. In this study, we found lico induced apoptosis and ER stress, and inhibited cell proliferation and colon formation dosage-dependently in H292 cells. Subsequently, manipulation of miR-144-3p level by transfecting either pre-miR-144 or siRNA/inhibitor substantiated that the level of miR-144-3p was crucial for the bioactivities of lico A, such as inhibition on proliferation and colon formation, as well as apoptosis/ER stress-inducing effects in H292 cells. Furthermore, the RT-qPCR result showed lico A could increase miR-144-3p level. The potential mechanism of natural products regulating miRNA expression involves interfering with pre-miRNA expression and modulating pre-miRNA dicing. In this study, pre-miR-144, which is the precursor of miR-144-3p, was used to overexpress miR-144-3p. And we observed a 50-fold increase in the miR-144-3p expression following the transfection of pre-miR-144. Meanwhile, in the pre-miR-144/lico A co-transfection group, the level of miR-144-3p was also significantly increased, compared with that in the pre-miR-144 transfection group. This result suggested that lico A increased miR-144-3p level through enhancing pre-miR-144 dicing, rather than increasing the pre-miR-144 level, for lico A is less likely be able to increase the pre-miR-144 level by over forty times as we found that lico A increase the level of miR-144-3p by around only 30%.

Besides clarifying the role miR-144-3p in lico A-induced ER stress, apoptosis and inhibitory effect on cell proliferation in H292 cells, another interesting phenomenon we found is that 40 μM lico A, though it could suppress H292 proliferation and colon formation more potently that 10 μM lico A, would not induce apoptosis as 10 μM lico A did in H292 cells. The only explanation for this is that higher level lico A inhibited certain target due to increased concentration, which means this inhibition would not be triggered until the concentration of lico A is high enough. As we known, lico A is the main component in the traditional Chinese medicine “licorice.” There has been a saying that if there were 10 prescriptions, nine of them must have licorice included. The daily dose of licorice was around 10–15 g, and the dose would be considered reasonable until 30 g per day. The content of lico A in licorice is around 4.68‰. So 40 μM of lico A still falls into the scope of clinical usage.

In order to fully understand the function of 40 μM lico A and the potential target that 40 μM lico A inhibited, first we evaluated if 40 μM lico A would still cause ER stress and autophagy. The endoplasmic reticulum (ER) is the organelle where transmembrane proteins and proteins are synthesized and folded. To be noted, ER is also crucial for other cellular functions, including Ca^2+^ buffering and the biosynthesis of phospholipids and cholesterol. And the aforementioned process of transmembrane protein, phospholipid and cholesterol synthesizing is ATP-demanding and thus depends on the right ionic strength. The compromise of many homeostatic processes would lead to the accumulation of unfolded or misfolded proteins in the ER lumen, which is known as ER stress. The ER stress activates the unfolded protein response (UPR), ER-associated degradation (ERAD) and autophagy that can help cells adapting to harmful stimuli under physiological conditions. The relationship between autophagy and cancer is complicated for different steps of autophagy have different roles in tumor generation and tumor survival (Mizushima, 2007). Total opposite results can be derived from different researches, focusing on the role of autophagy in cell fate under ER stress. Although autophagy was reported to have a pro-survival function following ER stress in most researches (Cheng et al., 2015), many studies proved an autophagy-mediated apoptotic process that would finally lead cancer cell to death (Huang et al., 2017).

There are three key signaling proteins activated in UPR, PKR-like endoplasmic reticulum kinase (PERK), inositol-requiring enzyme 1 (IRE1), and activating transcription factor 6 (ATF6). PERK is a sensor that phosphorylates α-subunit of the eukaryotic translation-initiation factor 2 (eIF2α) in ER, thereby activating eIF2α and reducing translation initiation and repression of protein synthesis (Ma et al., 2013). Activating transcription factor 4 (ATF4) and C/EBP homologous transcription factor (CHOP) are downstream genes of p-eIF2α. p-eIF2α selectively increases translation of ATF4 and its downstream gene CHOP expression (Harding et al., 2000). The overexpression of C/EBP-homologous protein (CHOP) results in B-cell lymphoma 2 (Bcl-2) down-regulation, BCL2-associated X protein (Bax) overexpression and enhanced translocation of Bax from the cytoplasm to the mitochondria, which ultimately leads to cell death through apoptosis (Gotoh et al., 2004).

The results showed that 40 μM lico A increased the PERK-CHOP pathway and caused autophagy in H292 cells, while the downstream genes of CHOP, such as apoptosis-related genes (caspase-3/8, Bcl-2, BAX, and BIM), were not activated, suggesting CHOP might be the key factor that 40 μM lico A targeted and inhibited. Using the 3D structure that we establish for CHOP protein (there is no crystal structure of CHOP available), docking studies were implemented and the results showed that lico A exhibited potent binding potentials in both the DNA-binding and dimer interfaces of CHOP, and therefore prevented the down-stream apoptotic factors from being activated.

In summary, we for the first time revealed that miR-144-3p was involved in the lico A-induced apoptosis, ER stress, and inhibition on proliferation and colon formation in H292 cells. The underlying mechanism was, at least partially, attributed to the enhancement of pre-miR-144 dicing and thereby miR-144-3p level by lico A. Moreover, 40 μM lico A could occupy and inhibit CHOP protein, and therefore prohibit the activation of its down-stream apoptotic factors.

## Author Contributions

GC, XL, and NL conceived and designed the experiments. YF, YH, and YT performed the experiments. YM and ZJ analyzed the data. GC, JZ, and HN wrote the paper.

## Conflict of Interest Statement

The authors declare that the research was conducted in the absence of any commercial or financial relationships that could be construed as a potential conflict of interest.
